# Supplementary figures and images for: Epidemiological, socio-demographic and clinical features of the early phase of the COVID-19 epidemic in Ecuador
Source: PLoS Negl Trop Dis. 2021 Jan 4;15(1):e0008958. doi: 10.1371/journal.pntd.0008958 (PMC7817051; doi:10.1371/journal.pntd.0008958)

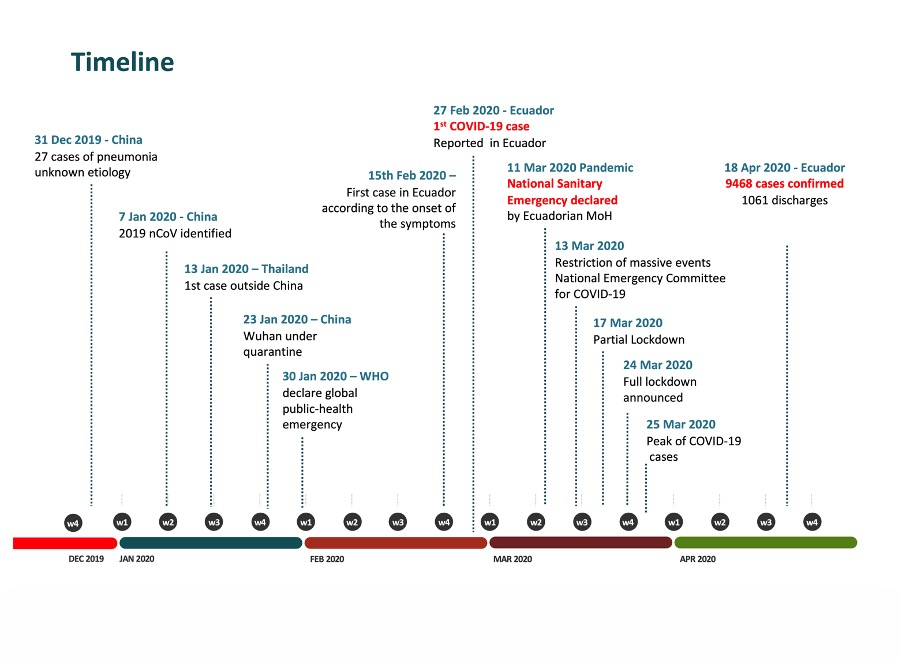

Supplement: S1 Fig — (TIF) [file pntd.0008958.s001.tif]

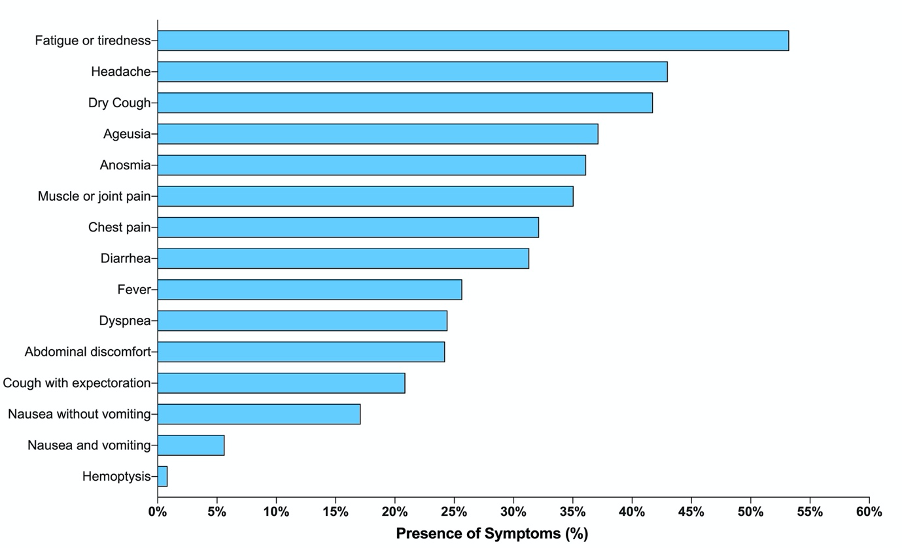

Supplement: S2 Fig — (TIF) [file pntd.0008958.s002.tif]

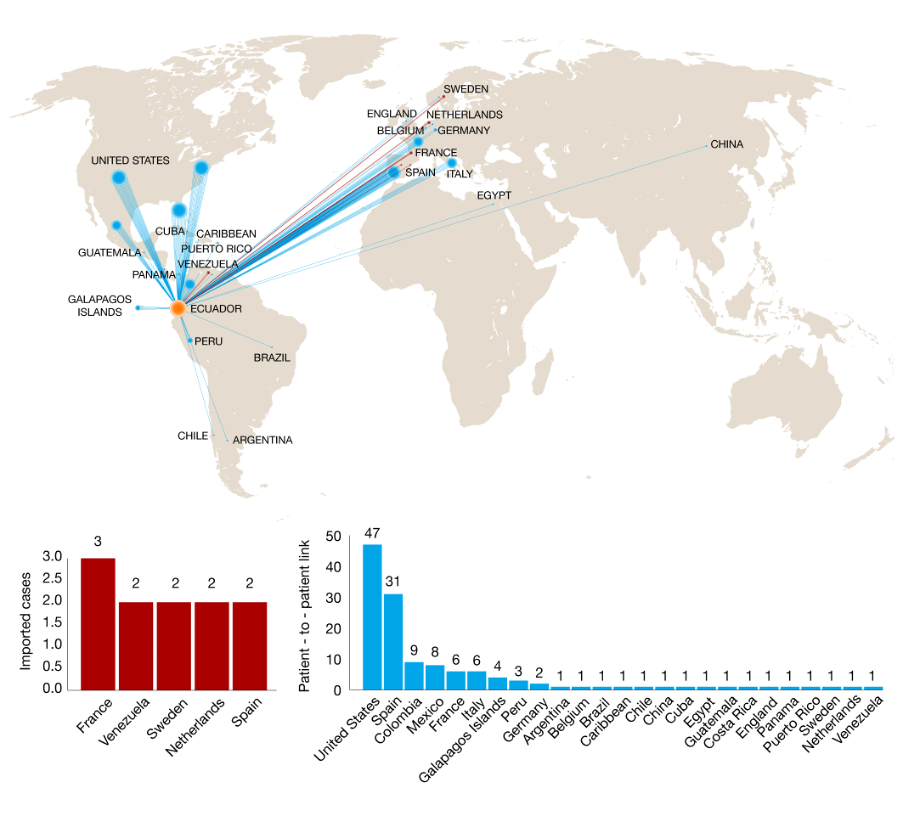

Supplement: S3 Fig — (TIF) [file pntd.0008958.s003.tif]

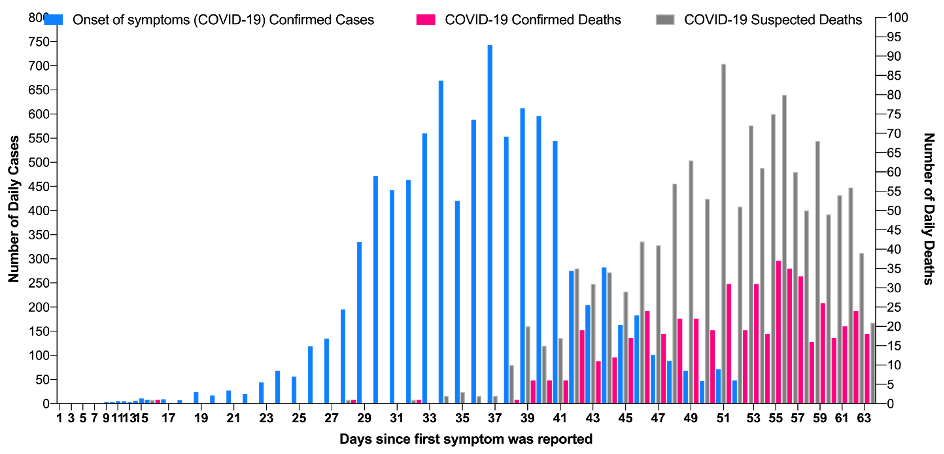

Supplement: S4 Fig — (TIF) [file pntd.0008958.s004.tif]

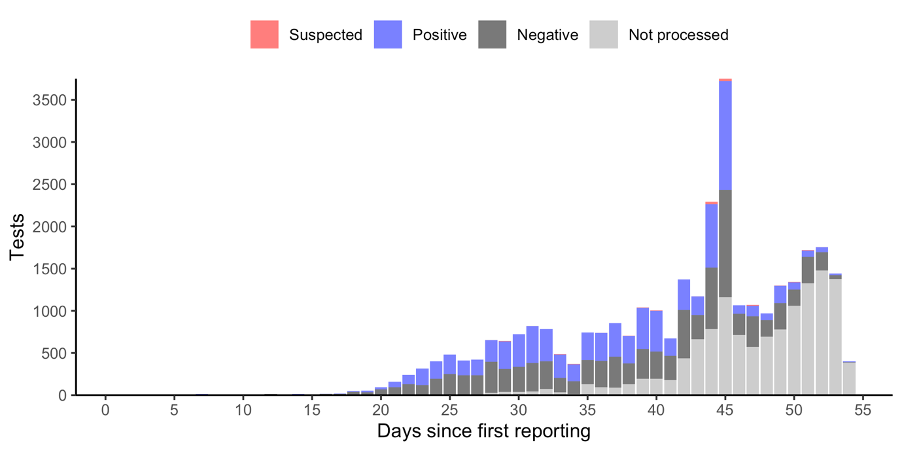

Supplement: S5 Fig — Number of RT-PCR tests performed since the day of first reporting (February 27th 2020) that were suspected (red), positive (blue), negative (black) and tests not processed (grey). (TIF) [file pntd.0008958.s005.tif]

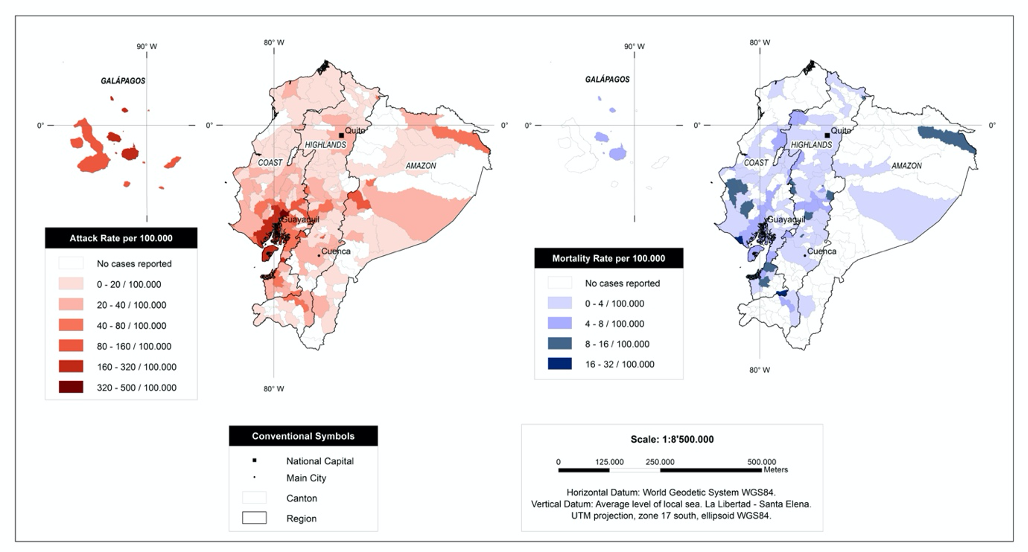

Supplement: S6 Fig — (TIF) [file pntd.0008958.s006.tif]

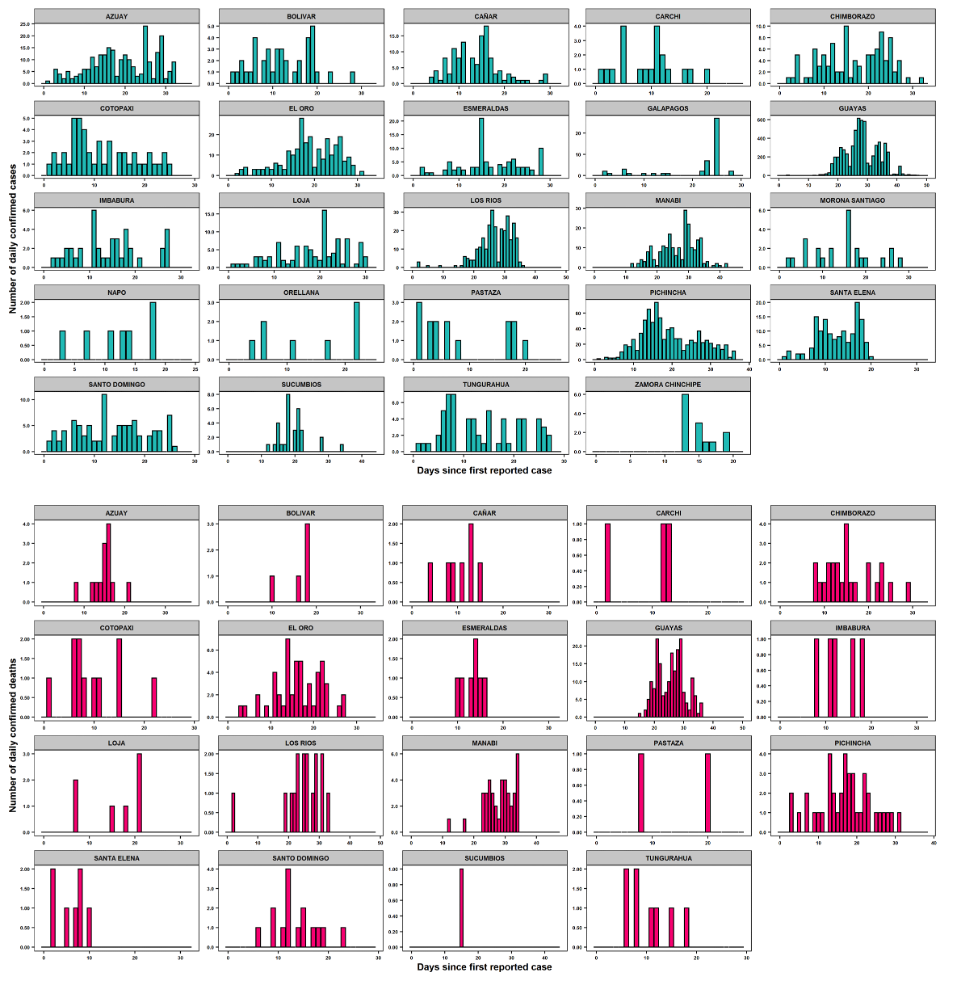

Supplement: S7 Fig — (TIF) [file pntd.0008958.s007.tif]
