# Supplementary material for: Epidemiological, socio-demographic and clinical features of the early phase of the COVID-19 epidemic in Ecuador
Source: PLoS Negl Trop Dis. 2021 Jan 4;15(1):e0008958. doi: 10.1371/journal.pntd.0008958 (PMC7817051; doi:10.1371/journal.pntd.0008958)
Supplement: S1 Table — Number of suspected, negative and positive tests performed, and positive testing rates (PTR%). (DOCX) [file pntd.0008958.s008.docx]

# Supplementary Tables

S1 Text*.* RT-PCR testing capabilities in Ecuador. Number of suspected, negative and positive tests performed, and positive testing rates (PTR%).

| **Laboratory** | **Suspected** | **Negative** | **Positive** | **Total** | **PTR%** |
| --- | --- | --- | --- | --- | --- |
| INSPI Cuenca | 0 | 1,548 | 548 | 2,096 | 26% |
| INSPI Guaranda | 0 | 14 | 17 | 31 | 55% |
| INSPI Guayaquil | 0 | 2,858 | 5,073 | 7,931 | 64% |
| INSPI Loja | 0 | 59 | 53 | 112 | 47% |
| INSPI Portoviejo | 0 | 0 | 1 | 1 | 100% |
| INSPI Quevedo | 0 | 0 | 2 | 2 | 100% |
| INSPI Quito | 9 | 2,149 | 1,028 | 3,186 | 32% |
| INSPI Santa Elena | 0 | 0 | 2 | 2 | 100% |
| INSPI Babahoyo | 0 | 0 | 1 | 1 | 100% |
| Public Hospitals | 8 | 440 | 261 | 709 | 37% |
| Private Laboratories | 86 | 2,695 | 2,441 | 5,222 | 47% |
| ABG/UDLA Lab | 0 | 541 | 41 | 582 | 7% |
| **Total** | 103 | 10,304 | 9,468 | 19,875 | 48% |
