# Supplementary material for: Epidemiological, socio-demographic and clinical features of the early phase of the COVID-19 epidemic in Ecuador
Source: PLoS Negl Trop Dis. 2021 Jan 4;15(1):e0008958. doi: 10.1371/journal.pntd.0008958 (PMC7817051; doi:10.1371/journal.pntd.0008958)
Supplement: Attachment — Submitted filename: POINT BY POINT.docx [file pntd.0008958.s010.docx]

**Point by point letter PLOS Neglected Tropical Diseases**

Dear Victoria J.Brookes,

Deputy Editor

PLOS Neglected Tropical Diseases

We would like to thank for the opportunity to submit a revised version of the manuscript entitled “Epidemiological, socio-demographic and clinical features of the early phase of the COVID-19 epidemic in Ecuador” for consideration for publication in PLOS NTD.

We would also like to thank the reviewers for the comments made on the previous version of the manuscript. These comments have helped us to increase substantially the quality of the paper.

Please find attached our point by-point response letter to the reviewer’s comments.

Reviewer #2:

1) Describe the epidemiological surveillance system in Ecuador. Where were the cases diagnosed? It seems that most of the cases were severe enough that required medical attention/hospitalization? Were any cases asymptomatic at the time of testing?

We have included the following paragraph (Page 9, Line 153-159):

The epidemiological surveillance system and contact tracing strategy denoted a lack of planning and the lack of enough personnel and resources. At the begging of the outbreak, every confirmed patient was followed and tracked by the health authority, nevertheless, when the number of new COVID-19 cases began to increase exponentially, the system’s capacity was outrunning. At the same time, testing capacities were collapsed and only those patients who presented symptoms, those who were in contact with any positive case or those lucky enough to get tested under the surveillance program obtained the diagnosis.

2) Could you add information about the testing rates over the course of the epidemic? What was the positivity rate?

We have provided the following data and phrase (Page 8, line 160-162):

The positive testing rate % (PTR%) was 43.19% (95%CI: 37.37-49.01) during the entire period of the study. In March 2020 the PRT% reached a mean of 39.90 (95%CI: 32.83-46.97), meanwhile in April, the PTR% was 44.94% (95%CI: 39.03-50.86).

Reviewer #1: Some issues with data presentation are noted in General Comments

Reviewer #2: 4) Since authors have the epi curve by date of symptoms onset, they could estimate the early reproduction number and assess the impact of interventions on the transmission rate. This addition would make the paper more interesting.

Thank you so much for the suggestion, we have included the following paragraph (Page 7-8, Line 153-159):

We calculated the basic reproduction number (R0) for the entire country using the exponential growth criteria. During the first months of the pandemic while numbers were increasing exponentially, the R0 was found to be 3.54 (CI: 3.46-3.63). When computed the R0 by provinces, we found R0 values as high as 3.68 (CI: 3.18-4.28) in Los Rios, followed by 3.68 (CI: 3.56-3.78) in Esmeraldas and 3.67 (CI: 3.56-3.78) in Guayas, while other provinces, especially those located in the highlands had significantly lower R0 values, including Pichincha with 1.28 (CI: 1.23-1.33), Azuay with 1.36 (CI: 1.24-1.49), and Loja 1.84 (CI: 1.57-2.16).

Reviewer #1:

In their manuscript, Ortiz-Prado et. al. present a cross-sectional analysis of the initial cases of COVID-19 in Ecuador. The data is interesting and provides insight into the trend of COVID-19 early in the transmission in South America, a region that has had a distinct lack of data transparency thus far. Overall, the manuscript tis useful and the data is clear; however, several issues and comments should be addressed and limitations need to be fully considered.

Thank you so much for your comments, they are greatly appreciated. We believe this is truly important to point out since after few weeks of the competition of this manuscript, data stopped to been shared with researchers and this is a unique opportunity to understand the real impact of the pandemic in Ecuador.

1) Please consider a full edit for English tense, grammar, and syntax to remain consistent throughout the entire manuscript.

Thank you very much for your comments, we have revised the manuscript entirely.

2) Please remove statements of primacy.

This recommendation was accepted, and the statement removed from the manuscript.

3) Introduction: As the pandemic is a very dynamic situation, please qualify COVID-19 numbers with a respective date (and update to most recent data available) in order to maintain accurate reporting in publication over the current global crisis.

We have included your suggestion and the data were updated until September 3th 2020. (Page 3, line 58-60)

4) Introduction: airborne transmission and it’s association with SARS-Co-2 spread is still under debate (though data does indicate it is possible). Please also consider using the term “droplet transmission.”

We have added your Recommendation (Page 3, line 61).

5) Please indicate how individuals were diagnosed and confirmed with SARS-CoV-2 infection.

All the cases were diagnosis using reverse transcription polymerase chain reaction (RT-PCR) technique. Your Recommendation was added in the manuscript (Page 3, line 68-69).

6) Figure 1: Only 143 individuals are reported in this figure? How can community-transmitted cases have a geographical origin other than Ecuador? Overall, this figure is a bit confusing and is not discussed in the text; therefore, it may be better to discuss or to leave out entirely.

We have updated the manuscript and the figure was removed.

7) Figure 2: blue and purple colors do not translate to grayscale, please consider revising

The figured was modified accordingly.

8) Based on the limited power, (n=84) for vaccinated individuals, it may not be advisable to indicate that mortality risk was lower in this population, especially since statistical significance was not achieved.

This analysis was removed, and just the data were described (Page 6, line 121-124).

9) Figure 3 is marked as Figure 1 in the legend. Please correct.

We have changed it.

10) As the pandemic continues, and Ecuador is not experiencing >70,000 cases of COVID-19 wirh no indication of curve reduction: it may be better to refer to all data “during the given time period” or with the specific dates as to ensure accurate usage of the facts and figures calculated.

We have added your suggestion to the manuscript

11) Is the comparison between the coastal and highland regions due to any issues of bias in the reported data or how the virus was introduced into the country and spread?

We proposed to establish this difference between regions due to the hypothesis of the existence of a possible relationship between COVID-19 mortality and high altitude reported by Reyes at al, 2020. Ecuador is one of the very few countries in the world that has populations living at elevations ranging from 0 to 4,500 meters, therefore we believe that showing these differences might be relevant for the readers. In this sense, we have added the following paragraph within the discussion.

In terms of elevation we found that people living in cantons and provinces located in the highlands had lower attack rates and lower mortality rates than the populations living at lower altitudes. We suggest that the virus spreads faster at lower altitudes due to demographic and cultural differences. We hypothesized that harsh weather (cold and windy) invites people to stay indoors while at sea level and due to warmer tropical climate, people tend to be more socially active and have greater mobility than the highlands. Additionally, demographic density is lower in the highlands according to the latest INEC data in Ecuador, situation that might influence the speed of the spread in terms of viral transmission. (Page 11, Line 267-273)

12) Discussion: may want to modify the statement about Ecuador and Panama affectedness with the current situation in Brazil, etc…again, it is understandable that these rankings and figures are extremely difficult to keep up with in terms on an ongoing global pandemic.

We have updated the information and included those countries with higher attack rates during the last weeks. (Page 8, Line 186-188)

13) While the lack of data on comorbidities and presence of clinical manifestations does represent a significant limitation, there are a number of other equally important limitations set out in the discussion (ie: information on testing and access to healthcare) and others which are not discussed (ie: unknown climate, viral, or social factors) which are also equally important. It may be better to restructure the discussion to address all possible limitations in a highly succinct way.

We have expanded in limitation section (Page 12, Line 282-289)

14) Please put consider including statistical results in Table 1, thus mitigating the need for Table 2 and making data comparison easier to analyze.

Table 2 was removed and the analysis was already described in a new paragraph within results section. (Page 6, line 125-128).

Reviewer #2: In this paper authors present the results of an interesting descriptive analysis of the epidemiological characteristics of a series of confirmed cases with COVID-19 in Ecuador. Overall the paper is well written and the findings reported here add to our understanding of the impact of the pandemic in Latin America and beyond. However, I have a few comments that need to be addressed in a revised version:

1) Describe the epidemiological surveillance system in Ecuador. Where were the cases diagnosed? It seems that most of the cases were severe enough that required medical attention/hospitalization? Were any cases asymptomatic at the time of testing?

This comment have already been responded in a new paragraph (Page 9, line 195-201):

The epidemiological surveillance system and contact tracing strategy denoted a lack of planning and the lack of enough personnel and resources. At the begging of the outbreak, every confirmed patient was followed and tracked by the health authority, nevertheless, when the number of new COVID-19 cases began to increase exponentially, the system’s capacity was outrunning. At the same time, testing capacities were collapsed and only those patients who presented symptoms, those who were in contact with any positive case or those lucky enough to get tested under the surveillance program obtained the diagnosis.

2) Could you add information about the testing rates over the course of the epidemic? What was the positivity rate?

This comment have already been responded (Page 8, line 160-162):

The positive testing rate % (PTR%) was 43.19% (95%CI: 37.37-49.01) during the entire period of the study. In March 2020 the PRT% reached a mean of 39.90 (95%CI: 32.83-46.97), meanwhile in April, the PTR% was 44.94% (95%CI: 39.03-50.86).

3) Authors need to put their findings in context. There are now a few studies reporting key transmission and epidemiological characteristics (including the adjusted CFR and the reproduction number) of the pandemic impact in Latin American countries including Brazil, Peru, and Chile.

Thank you for pointing this out, we have included some of the references Nº 40-43. (Page 11, line262-266)

4) Since authors have the epi curve by date of symptoms onset, they could estimate the early reproduction number and assess the impact of interventions on the transmission rate. This addition would make the paper more interesting.

This comment have already been responded.

5) Authors should update the analysis using the most recent data available.
The importance of our analysis is based on the fact that after April 20, the data that was included in our study was removed from the official freely accessible databases and even though several months have passed, most of the information is still not publicly available.
