# Supplementary material for: Epidemiological, socio-demographic and clinical features of the early phase of the COVID-19 epidemic in Ecuador
Source: PLoS Negl Trop Dis. 2021 Jan 4;15(1):e0008958. doi: 10.1371/journal.pntd.0008958 (PMC7817051; doi:10.1371/journal.pntd.0008958)
Supplement: Attachment — Submitted filename: POINT BY POINT third review docx.docx [file pntd.0008958.s011.docx]

**Point by point letter PLOS Neglected Tropical Diseases**

Dear Victoria J.Brookes,

Deputy Editor

PLOS Neglected Tropical Diseases

We would like to thank for the opportunity to re-submit a revised version of the manuscript entitled “Epidemiological, socio-demographic and clinical features of the early phase of the COVID-19 epidemic in Ecuador” for consideration for publication in PLOS NTD.

We would also like to thank the reviewers for the comments made on the previous version of the manuscript. These comments have helped us to increase substantially the quality of the paper.

Please find attached our point by-point response letter to the reviewer’s comments.

Please incorporate the complete methodology and additional results into the methods and results in the main paper.

We have added the complete methodology and the additional results in the main manuscript. We have also organized the results section, accordingly, adding subheadings, tables and figures

Line 67 ‘covid19’ to ‘COVID-19

Thanks, we have changed it to COVID-19

Line 105 Please re-write this sentence because the meaning is not clear:

‘We computed the time distribution from hospital admission to the time of using the methodology reported by Russel 2020.’

We have reviewed the sentences and we have changed to “We have adjusted the naive estimates of CFR% to account for the delay from hospitalization-to-death for those cases that were fatal using a delay-adjusted case fatality ratio proposed by Russel et.al 2020.”

Line 131: Please check the figure numbers throughout the manuscript.

We have corrected the figure numbers throughout the entire manuscript

Line 139:

‘These differences were compared with the national distribution for each ethnic group according to the 2010 national census.’

Where are the results for these comparisons? If this is included in Table 1, please make this clear (here, and in the Table labels).

Thanks for your comment, we have rewritten the sentence and moved it to settings within the methodology section as follow:

Ethnicity in Ecuador is distributed as follows: 7.1% indigenous (1,059,863), 7.2% Afro Ecuadorian (1,077,878), 79.3% Mestizo (11,910,702), 6.1% self-reported white or Caucasians (909,741) and 0.4% other groups according to the 2010 national census^10^.

Line 146: ‘Had’ to ‘have’

We have corrected the entire section and the mistake was replaced.

Line 157: The meaning of this sentence is not clear. Please re-write and check the figure number (for previous figures too – Figures 1 and 2 appear to be labelled the wrong way round).

We have observed the mistake and rearranged the sentence as follow:

The median time elapsed between the onset of symptoms and the time before receiving medical attention was four days (IQR: 1-8 days).

‘When 158 observed retrospectively using the date of the onset of symptoms, 29 undocumented patients were already sick but only 6 were diagnosed, trend that continued to be high until March 24th 159 , 160 the day when the lock-down was implemented in Ecuador (Figure 2).’

The labeling if figures was amended

Line 211:

‘At the beginning of the pandemic, Ecuador registered a single case on February 27, when in 212 reality, looking retrospectively, there were already 19 undetected cases. Only two weeks later, 213 there were 11 officially registered cases but at least 119 undetected cases (Figure 3).’

We have fixed the typo and corrected the sentence as follow: …..29 undetected cases. Only two weeks later, only 11 cases were officially registered, but data shows that at least 119 cases were actually circulating within the country (Figure 3).

Where is Figure 3, and please explain more clearly how numbers of undetected cases were known (if numbers are known, was this retrospective case ascertainment?). If these are the results of this analysis, move this sentence and explanation to the results.

Figure 3 is now correctly labeled, and the number of cases was known by; interestingly, they used the date of diagnosis instead if the date of the initial symptoms; that is why this report is the first one pointing out these findings, data obtained directly from the database that was shared by the MoH.

Line 217:

Please clarify this sentence (‘was denoted by’?) and state how this is known (is it inferred from the findings of this study, or other evidence?): ‘The epidemiological surveillance system and contact tracing strategy denoted a lack of planning and the lack of enough personnel and resources.’

Thanks, so much for your comment, we have rewritten the sentence as follow:

According to the high PTR% found in Ecuador, the excess mortality that was not correctly diagnosed and reported as COVID-19 and other variables; we inferred that the epidemiological surveillance system and contact tracing strategies were not correctly implemented

Line 218 ‘begging’ to ‘beginning’

The typo was corrected

Line 279: ‘calculation age-specific attack’ to ‘calculation of age-specific attack’

Thanks, so much for your comments, the suggestion was included.

Line 295: ‘a situation’

We have added your suggestion
