# Supplementary material for: Epidemiological, socio-demographic and clinical features of the early phase of the COVID-19 epidemic in Ecuador
Source: PLoS Negl Trop Dis. 2021 Jan 4;15(1):e0008958. doi: 10.1371/journal.pntd.0008958 (PMC7817051; doi:10.1371/journal.pntd.0008958)
Supplement: Attachment — Submitted filename: POINT BY POINT 4th review docx.docx [file pntd.0008958.s012.docx]

**Point by point letter PLOS Neglected Tropical Diseases**

Dear Victoria J.Brookes,

Deputy Editor

PLOS Neglected Tropical Diseases

We would like to thank for the opportunity to re-submit a revised version of the manuscript entitled “Epidemiological, socio-demographic and clinical features of the early phase of the COVID-19 epidemic in Ecuador” for consideration for publication in PLOS NTD.

We would also like to thank the reviewers for the comments made on the previous version of the manuscript. These comments have helped us to increase substantially the quality of the paper.

Please find attached our point by-point response letter to the reviewer’s comments.

There are still some remaining unusual items in the supplementary material. Finally please update as follows:

Additional Conclusions - to manuscript

done

Acknowledgement – remove

done

Authors' Contributions - can leave

done

Disclosure Statement – remove

done

Funding Sources - remove

done
